# Quantitative propagation of assembled human Tau from Alzheimer's disease brain in microfluidic neuronal cultures

**DOI:** 10.1074/jbc.RA120.013325

**Published:** 2020-07-22

**Authors:** Antigoni Katsikoudi, Elena Ficulle, Annalisa Cavallini, Gary Sharman, Amelie Guyot, Michele Zagnoni, Brian J. Eastwood, Michael Hutton, Suchira Bose

**Affiliations:** 1 Department of Neuroscience, Eli Lilly & Company Limited, Erl Wood Manor, Windlesham, Surrey, United Kingdom; 2 Centre for Microsystems & Photonics, Department of Electronic and Electrical Engineering, University of Strathclyde, Glasgow, United Kingdom

**Keywords:** human, microtubule, neurodegeneration, protein aggregation, Tau protein (Tau), tauopathy, drug screening, Alzheimer disease, neurodegenerative disease, neuron, microfluidics, propagation

## Abstract

Tau aggregation and hyperphosphorylation is a key neuropathological hallmark of Alzheimer's disease (AD), and the temporospatial spread of Tau observed during clinical manifestation suggests that Tau pathology may spread along the axonal network and propagate between synaptically connected neurons. Here, we have developed a cellular model that allows the study of human AD-derived Tau propagation from neuron to neuron using microfluidic devices. We show by using high-content imaging techniques and an in-house developed interactive computer program that human AD-derived Tau seeds rodent Tau that propagates trans-neuronally in a quantifiable manner in a microfluidic culture model. Moreover, we were able to convert this model to a medium-throughput format allowing the user to handle 16 two-chamber devices simultaneously in the footprint of a standard 96-well plate. Furthermore, we show that a small molecule inhibitor of aggregation can block the trans-neuronal transfer of Tau aggregates, suggesting that the system can be used to evaluate mechanisms of Tau transfer and find therapeutic interventions.

Alzheimer's disease (AD) is the most common cause of dementia, a neurological disorder that is currently believed to affect 35.6 million people worldwide and estimated to triple by 2050. A key neuropathological hallmark of AD and other tauopathies is the abnormal folding and hyperphosphorylation of Tau protein, which leads to generation of Tau filaments and neurofibrillary tangles. During the clinical manifestation of AD, a temporospatial spreading of Tau-positive neurofibrillary lesions is observed, suggesting that once Tau pathology is initiated it may spread along the axonal network and propagate between connected neuronal cells; moreover, the extent of Tau pathology strongly correlates with symptom severity and neuronal cell loss (1). To classify Tau pathology in AD, Braak and Braak (1) developed a six-tiered system of disease staging based on silver-stained, hyperphosphorylated Tau aggregates (1–3). According to this staging system, hyperphosphorylated Tau accumulates first in the entorhinal cortex and locus coeruleus before the disease becomes symptomatic; the propagation of Tau pathology beyond entorhinal cortex and locus coeruleus by neuron-to-neuron transmission is initiated after accumulation of a high β-amyloid load in isocortical regions and is associated with symptoms of AD, according to pathological, clinical, and biomarker data (4). This temporal and spatial pattern of spreading observed in tauopathies supports the theory of trans-synaptic spreading of Tau, which has also been demonstrated *in vivo* and in cell culture models (5–8). However, alternative, nonsynaptic transfer of Tau between synaptically connected neurons has not been excluded (9–15). Cellular and animal models recapitulating features of tauopathies provide a useful tool to investigate the causes and consequences of Tau aggregation. Cellular models are useful for understanding disease mechanisms, screening, and profiling compounds that interfere with Tau aggregation. Microfluidic devices represent a miniaturized alternative to recapitulate disease conditions (16). These devices were first created to study axonal damage and repair (17), but their rapid development has now received attention from multiple scientific fields. Typically, to facilitate neuronal culture, microfluidic devices are fabricated using gas-permeable silicone elastomer polydimethylsiloxane (PDMS). The most common device layout used to create synaptically connected but environmentally isolated neuronal populations (18, 19) comprises two microfluidic culture compartments where neuronal cells grow processes and subsequently become connected by an array of microchannels through which axonal growth is guided; whether axons or dendrites can reach the opposite culture compartment depends on the length and shape of the microchannels (17, 20, 21). This structure enables axons from one culture compartment to form synaptic connections with dendrites from the other compartment. The main positive features of the microfluidic platform are small reaction volumes, leading to minimal reagent usage, and the control over spatial and temporal separation of neuronal populations, which allows simulation of a neuronal network (22). Because one of the main pathophysiological characteristics of AD is neuronal cell death with loss of synapses and neuronal network within the brain, microfluidic devices provide an ideal platform to study neuronal connectivity and spread of Tau pathology. Over the past two decades, microfluidics technology has significantly advanced, and microfluidic devices have now been employed in multiple publications to model neuronal networks and mimic Tau spreading in human tauopathies (7, 23–28). However, quantification of Tau pathology and spreading in these models was challenging, the robustness of the signal window could not be determined, and the throughput of the assays was minimal. Here, we designed microfluidic plates so that the user could handle 16 two-chamber devices simultaneously in the footprint of a standard 96-well plate, thus increasing throughput of information and facilitating interfacing with high-content imaging (HCI) equipment. We used primary nontransgenic rat cortical neurons (RCNs) seeded with human (h) AD Tau as a model for human sporadic tauopathies and were able set up a quantifiable and reproducible imaging assay in a medium-throughput microfluidic format for detecting formation and propagation of Tau aggregates. This model system could be ideal for testing the effect of potential Tau therapeutics that modulate Tau trans-neuronal propagation. Furthermore, we believe this to be the first time that a robust, reproducible methodology has been established for quantifying aggregates in microfluidic chambers, because this technology has previously struggled with delivering quantitative outcomes. This is despite the broad and increasing use of this technology. Protein aggregate propagation is central to many neurodegenerative disease progressions; therefore, the method can be further adapted to measure propagation of any prion-like disease.

## Results

### Purified seed from human AD brain seeds rodent Tau to form insoluble aggregates

**Figure 1.**
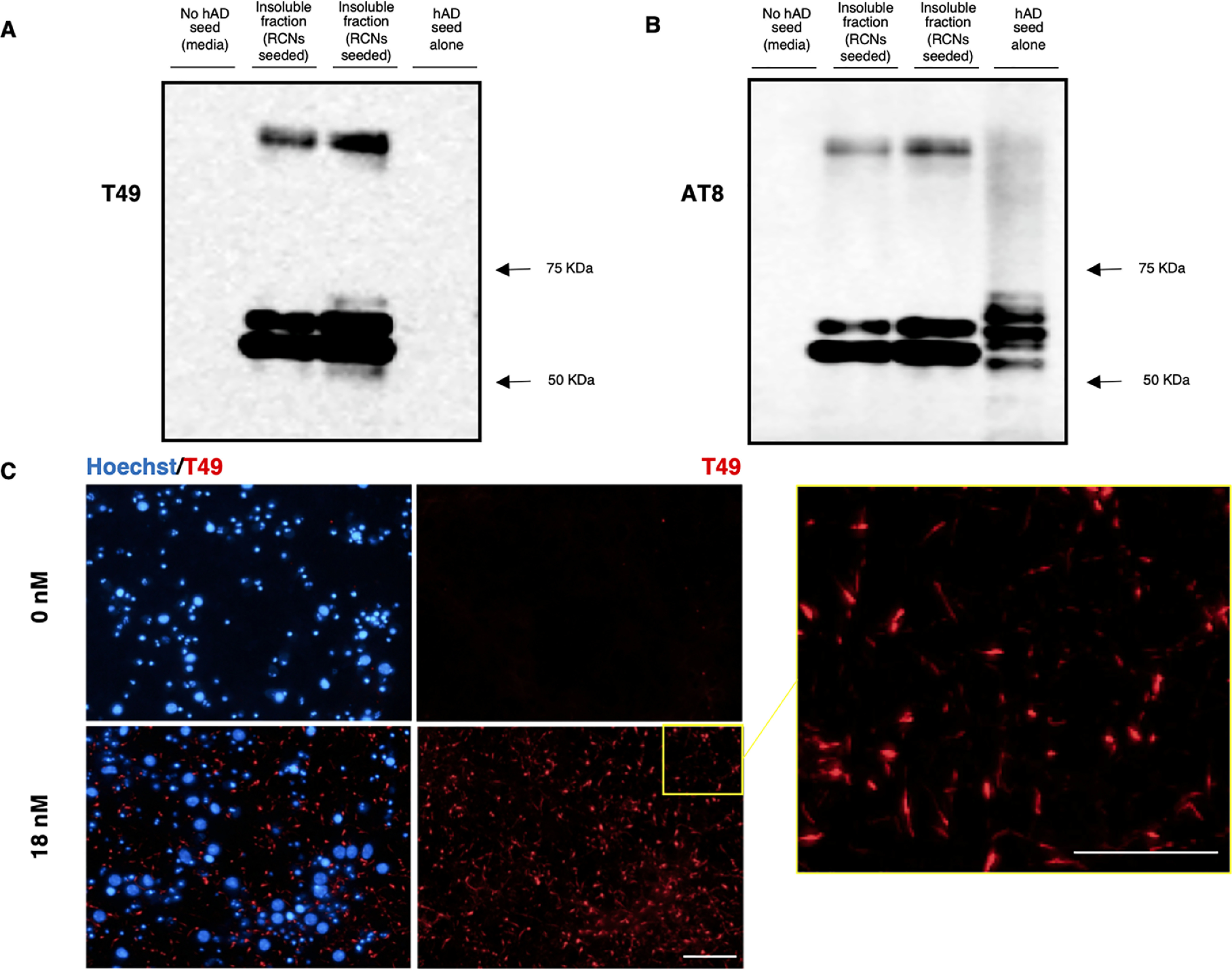
**Purified hAD seed recruits rodent Tau to form AT8 insoluble aggregates.** *A* and *B*, Western blotting of Sarkosyl extracted material from RCNs treated with hAD seeds (from two separate seeding extractions) and probed with rodent specific antibody, T49 (*A*) or pS202/205 Tau, AT8 (*B*). *C*, immunostaining of rodent Tau (T49, *red*) and nuclei (Hoechst, *blue*) of RCNs either unseeded (*top panels*) or seeded with 18 nm of hAD seed (*bottom panels*) in 96-well plate cultures. The zoomed image of the seeded condition (*yellow square*) shows the thread-like shape of the endogenous aggregates. Image acquired with Operetta. *Figure bar*, 10 μm; *zoomed image bar*, 5 μm.

**Figure 2.**
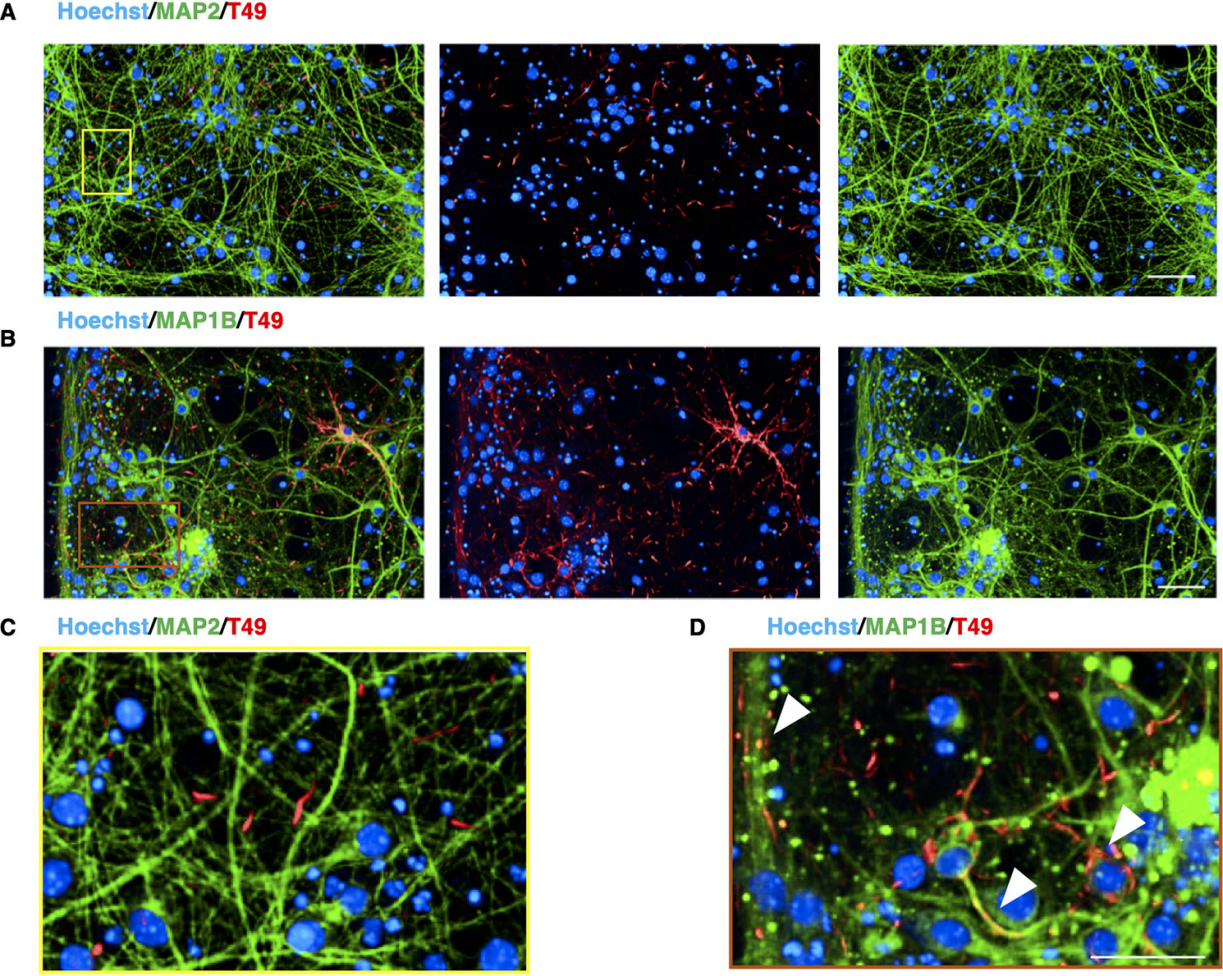
**T49-positive inclusions are localized in the axons and soma of RCNs.** *A–D*, sparse co-localization between the T49 neuritic thread-like inclusions in RCNs and MAP2, a specific dendritic marker (*A* and zoomed image in *C*), but good co-localization with MAP1B, a neuronal cytoskeleton marker (*B* and zoomed image in *D*), indicating that the inclusions observed after seeding with hAD seed are not dendritic but somatic and axonal. *Arrowheads* in the *D* indicate co-localization between MAP1B and T49. Images were acquired with Opera Phenix and 20× objective. *Figure bar*, 50 μm; *zoomed image bar*, 25 μm.

To extract seeding-competent material from h AD brains, we used a Sarkosyl extraction protocol adapted from Greenberg and Davies (29). This purified hAD seed was analyzed via AlphaScreen to determine Tau concentration, and 18 nm (optimal concentration as shown in Fig. S1) was added to RCNs at DIV 7; their cell lysate was biochemically characterized for Sarkosyl-insoluble Tau levels at DIV 21. Western blotting analysis of their Sarkosyl-insoluble pellet demonstrates that hAD seed when added to RCNs seeds rodent Tau as shown using the rodent specific antibody, T49 (Fig. 1*A*) that resulted in the formation of AT8-positive, Sarkosyl-insoluble Tau (Fig. 1*B*). We have analyzed 3R and 4R Tau expression in a time course in RCN (Fig. S2). The expression of 3R and 4R Tau increased over time, peaking at DIV 15 and then started to decline. We hypothesize that the endogenous aggregates that would template from hAD seed (starting from DIV 7) will contain both 3R and 4R Tau. By HCI we show that when hAD seed was added to RCNs at DIV 7 and the cells were fixed at DIV 21 and stained with the same rodent specific Tau antibody T49, the hAD seed could induce neuritic thread-like inclusions in a 96-well plate assay (Fig. 1*C*). In summary, hAD seed can seed rodent Tau to form neuritic thread-like inclusions that are comprised of Sarkosyl-insoluble 3R and 4R Tau, consistent with findings in mouse cortical neurons (30). Dendrite-specific MAP2 staining (Fig. 2, *A* and *C*) revealed no co-localization with T49 staining, whereas microtubule-specific MAP1B staining (Fig. 2, *B* and *D*) showed co-localization. Because the T49 inclusions did not co-localize with the dendrite-specific marker MAP2, we conclude that the T49-positive Tau inclusions are axonal and somatic because they co-localized with MAP1B, a neuronal marker that is present in axonal and somatic microtubules. In developing neurons, MAP1B and Tau are the main MAPs found in axons, whereas MAP2 is found in dendrites (31).

### Using microfluidic devices to study propagation of hAD templated rodent Tau

**Figure 3.**
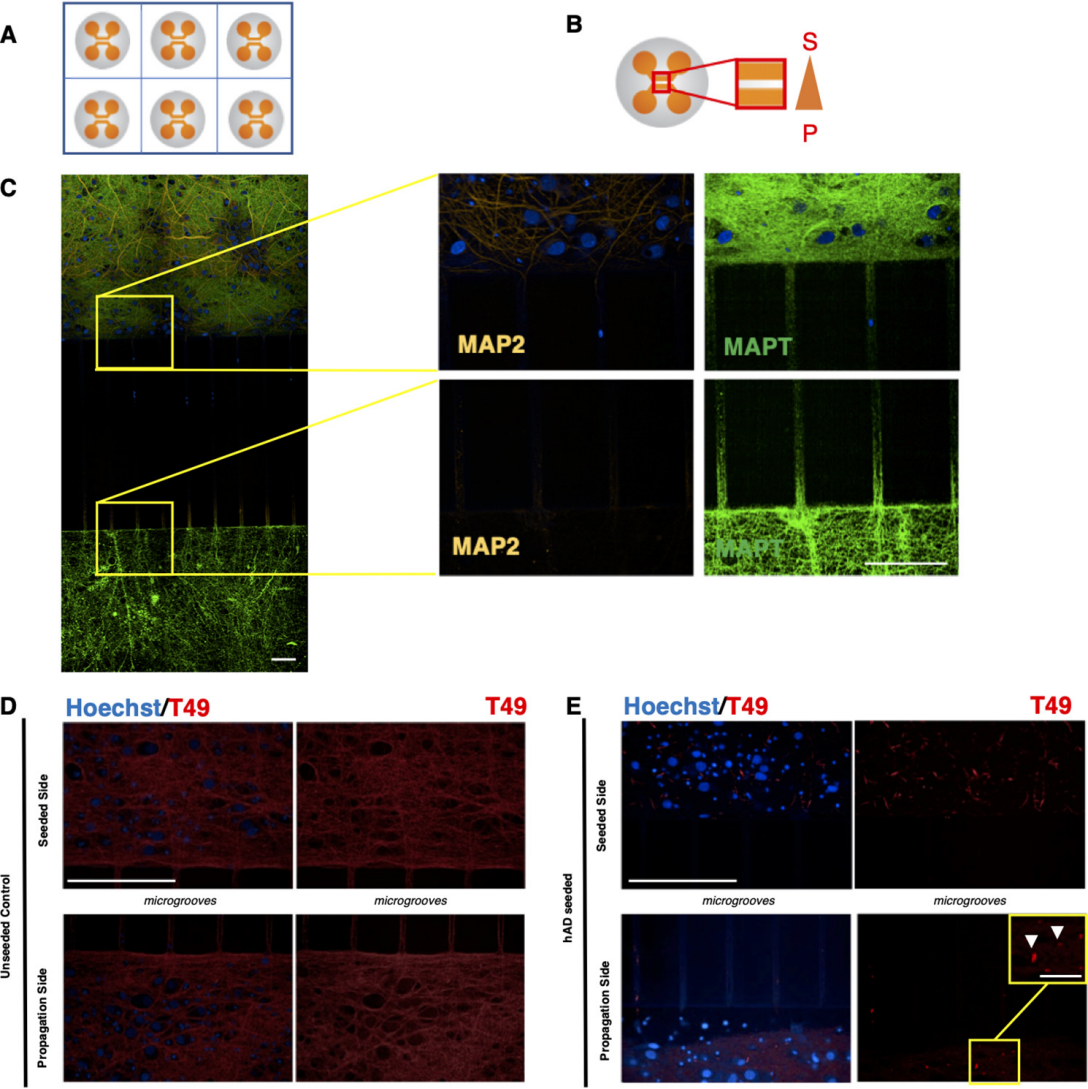
**RCNs were cultured in two-compartment microfluidic devices in a 6-well plate assay format to enable the study of trans-neuronal aggregated Tau.** *A*, schematic representation of a typical 6-well plate microfluidic assay format. *B*, the area of biological interest in a typical two-compartment microfluidic device is highlighted within the *red box*. To achieve fluidic isolation, the propagation side (*bottom chamber*, *P*) received almost 2-fold higher medium volume compared with the seeded side (*top chamber*, *S*). *C*, immunocytochemistry demonstrated that for RCNs cultured for 3 weeks in two-compartment microfluidic devices, only the axons (MAPT) can grow through the 450-μm-long microgrooves rather than the dendrites (MAP2). *Figure bar*, 15 μm; *zoomed image bar*, 50 μm. RCNs cultured in two-compartment microfluidic devices template the hAD seed and propagate. *D* and *E*, the control unseeded neurons represent a diffused T49 staining (*D*), whereas in the presence of the hAD seed (*E*), the neurons demonstrate neuritic thread-like inclusion morphology (*white arrows*). The results have been reproduced in two independent experiments. *Bar*, 50 μm. Image acquisition with Opera Phenix and 40× objective. Zoomed images are indicated by *yellow squares*. *Bar*, 12.5 μm.

**Figure 4.**
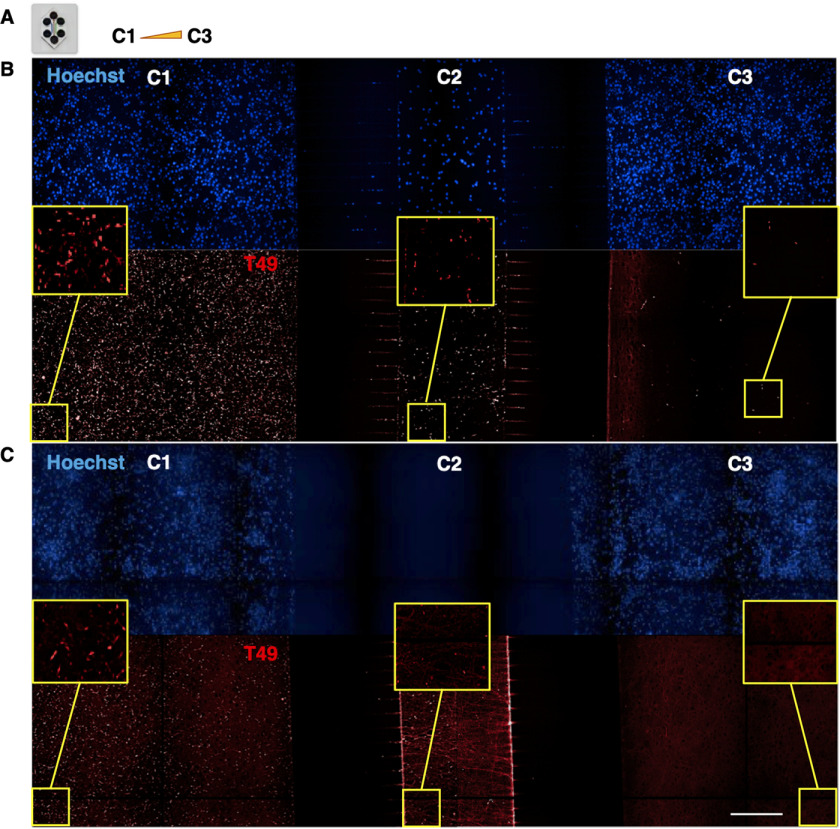
**Demonstration that propagation of endogenously aggregated rodent Tau is a real effect and not a device artifact in three-chamber microfluidic devices.** *A*, RCNs were plated on DIV 0 in compartments C1, C2, and C3 of a three-compartment microfluidic device with the volume gradient increasing from C1 to C3 and the neurons of C1 were seeded with 18 nm of hAD seed on DIV 7. *B*, the cells were fixed 2 weeks later following the timelines of the two-compartment microfluidic devices and stained for rodent specific Tau, T49. T49-positive inclusions were detected in C3. *C*, when no RCNs were plated in C2, there were no T49-positive inclusions detected in compartment C3, suggesting that there was no axonal growth overlap between the two neuronal populations. Image acquisition with Opera Phenix 20× magnification, image tiled montages with zooms for each compartment in the *yellow squares*. *Figure bar*, 50 μm; *zoomed image bar*, 12.5 μm.

We moved on to test whether our hAD seed preparation could seed RCNs cultured in two-compartment microfluidic devices. For our initial experimental setup, RCNs were cultured over the course of 3 weeks in two-compartment PDMS microfluidic devices that were irreversibly plasma-bonded on the glass surface of 6-well imaging plates (Fig. 3*A*). The hAD seed was added on the seeded side, and the cells were fixed in devices; this plate format was used for HCI to detect aggregated pathological Tau at the propagation side (Fig. 3*B*). What is characteristic about the microfluidic devices used is that only the axons (Fig. 3*C*, Microtubule Associated Protein Tau (MAPT) staining) can grow through the 450-μm-long microgroove barriers that separate the two compartments but not the dendrites (Fig. 3*C*, MAP2 staining). This supports the idea that microfluidic devices can be used as an *in vitro* cellular model to study trans-neuronal and axonal propagation of aggregated Tau and is consistent with previous literature reports with recombinant and transgenic fibrils in transgenic cellular models (27, 32). Here, we have demonstrated that seeding RCNs with hAD seed results in propagation of endogenously generated rodent Tau in a microfluidic two-compartment model as revealed by a higher objective magnification (Fig. 3, *D* and *E*). Characteristically, the morphology of the aggregated neuronal Tau formed after seeding RCNs with hAD seed resembled the neuritic thread-like pathology observed previously in mouse cortical neurons (30). The inclusions observed in the propagation side of the microfluidic devices exhibited the same neuritic thread-like pathology as observed in the seeded side (Fig. 3, *D* and *E*) and in the 96-well assay (Fig. 1*C*). Optimization of the seeding concentration for the hAD seed in 96-well RCN cultures revealed that 18 nm (0.75 μg/ml) is a concentration that provides a statistically significant signal window (Fig. S1). The parameters considered were the count of T49 neuritic thread-like inclusions and the hAD seed dose response. Concentrations beyond 18 nm of hAD seed demonstrated a saturation in the signal window and occasional cell toxicity (Fig. S1). During our immunocytochemistry protocol, we used methanol as previously described (30) to fix the cells and remove soluble proteins. We observed diffuse background staining, mostly evident in the unseeded cells in both compartments, which could represent residual soluble protein (Fig. 3, *D* and *E*). Furthermore, to determine whether the propagation observed is a real effect and not an artifact of the two-compartment model caused by the axonal growth being longer than the microgrooves distance, three-compartment microfluidic devices were used, keeping the experimental parameters consistent with the two-compartment model described above. When RCNs were plated in the first (C1), middle (C2), and third (C3) compartments, hAD seed was added to C1 on DIV 7, and cells were fixed on DIV 21, T49 staining revealed inclusions in C3 (Fig. 4*B*). When RCNs were plated only in C1 and C3, leaving the middle compartment C2 empty, T49 staining revealed no inclusions in C3 (Fig. 4*C*). Plating cells only in the outer chambers decreased the probability of forming synapses, suggesting that a lack of synapse formation is a barrier to propagation, consistent with findings by Calafate *et al.* (7). However, neuritic inclusions are observed in C2. This could be because axons that grow through the microgrooves (Fig. 3*C*) make the detection of neuritic inclusions obvious in C2. These control experiments showed that hAD seeding results in true propagation because there is no axonal overlap between neurons in C1 and C3.

### The propagation of neuritic thread-like inclusions is quantifiable in the microfluidic culture system

**Figure 5.**
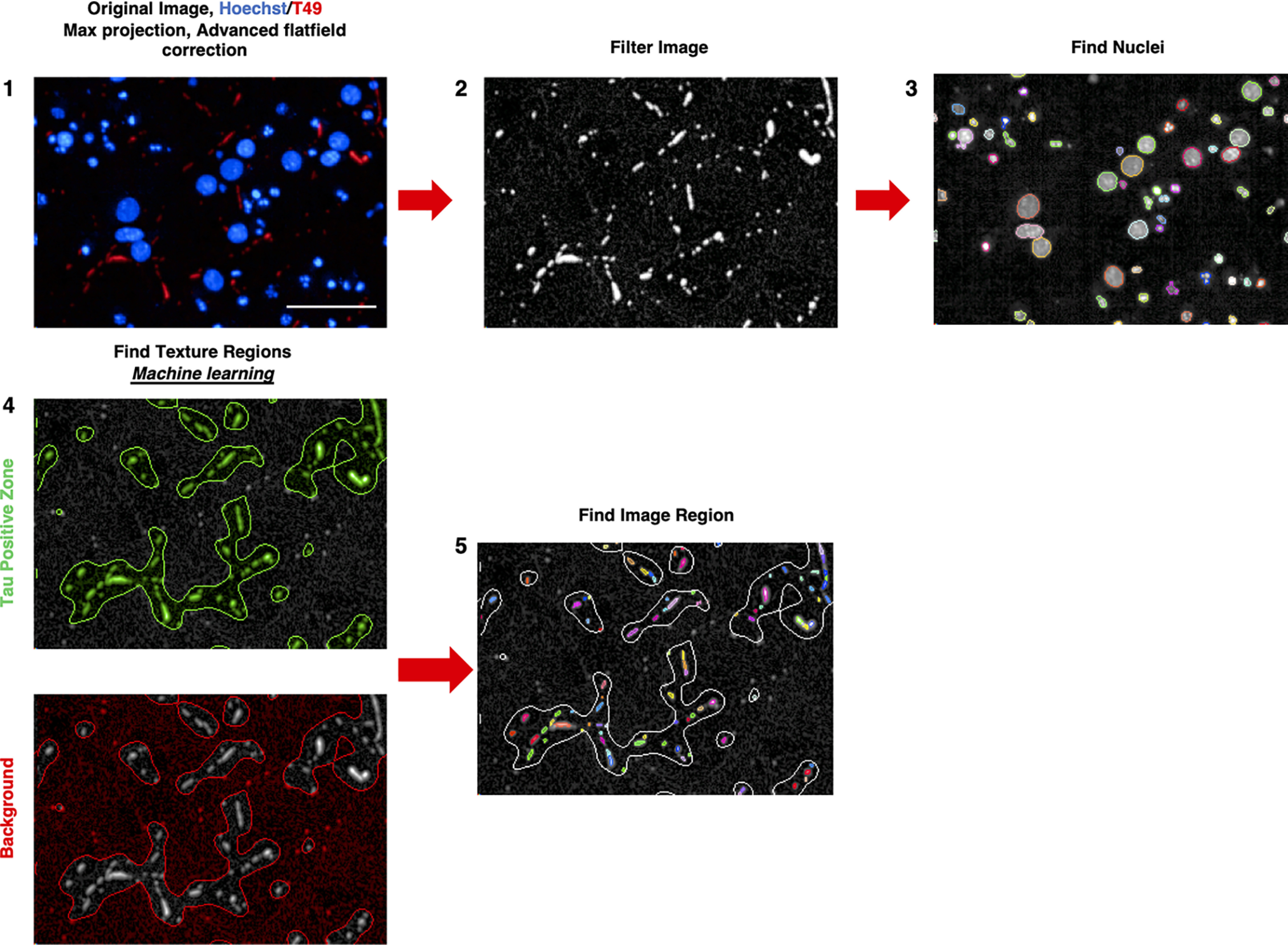
**High-content image analysis pipeline to quantify the number of T49 neuritic-like threads in a Tau-positive zone.** The original merged image undergoes maximum projection and flat-field correction using the Harmony software (*step 1*) before being filtered to eliminate and smooth the fluorescent background for the T49 staining (*step 2*). The total number of nuclei is counted with Hoechst (*step 3*), and the filtered T49 image undergoes machine learning to separate the background (unseeded condition) from the Tau-positive zone (T49 neuritic-like threads) (*step 4*). Finally, the number of T49 neuritic-like threads are counted within the Tau-positive zones (*step 5*). *Bar*, 50 μm.

**Figure 6.**
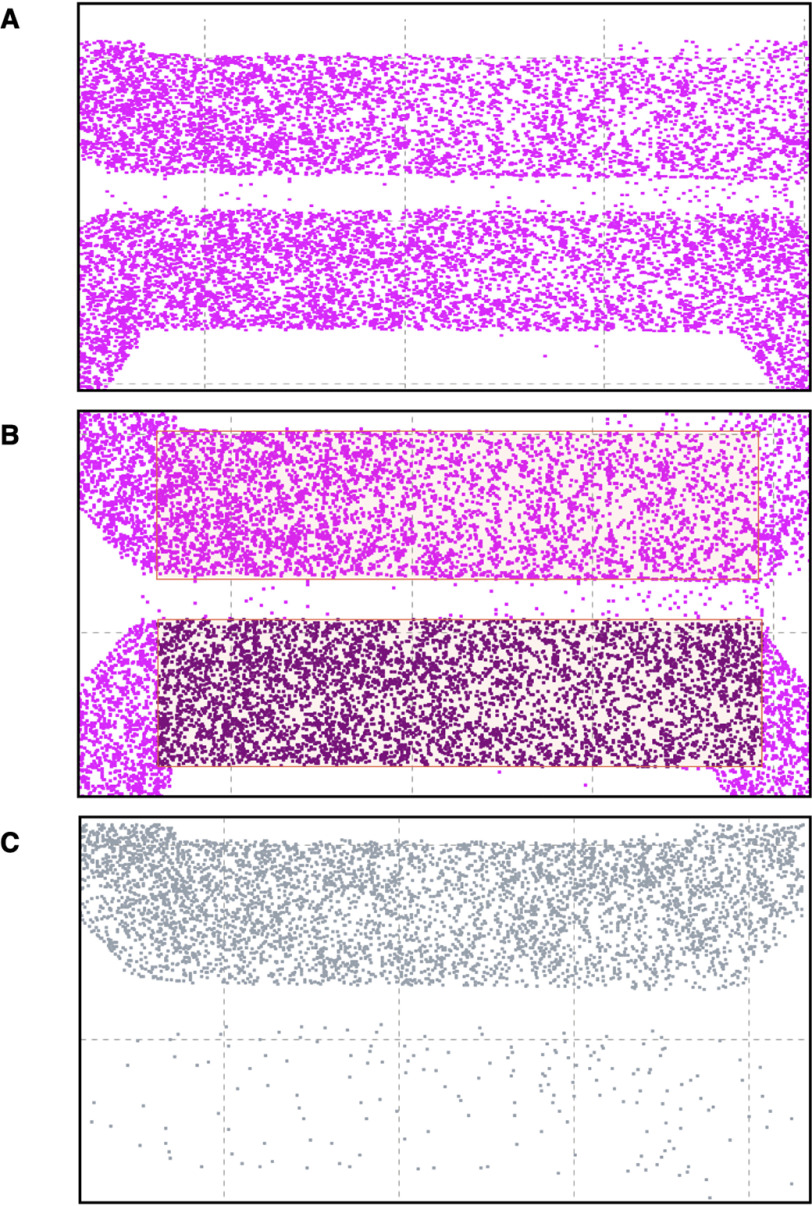
**A computer program for the visualization and processing of the T49 neuritic-like thread inclusions in the seeded (*top channel*) and propagation (*bottom channel*) side of two-compartment microfluidic devices.** Typical example of RCNs in a two-compartment microfluidic device. The cells were processed with HCI, and the results were loaded into the Cell Counter computer program. *A* and *B* illustrate the pixel visualization of the nuclei (Hoechst staining), and *C* shows the T49 neuritic-like threads in seeded (*top*) and propagation (*bottom*) compartments. The regions of interest in the *top* and *bottom* are auto-selected and processed for the number of nuclei and T49 neuritic-like threads by the program, but it also allows for human review and override by exception.

Although the propagation of Tau fibrils has been demonstrated before, the evidence has been qualitative rather than quantitative. To demonstrate the robustness of our microfluidic assay, we developed a novel high-content imaging pipeline to quantify the propagation of hAD-seeded rodent Tau inclusions in our microfluidic devices. Fig. 5 summarizes the main steps of the Harmony software analysis pipeline used to quantify the neuritic thread-like inclusions when RCNs in two-compartment microfluidic devices were seeded with the hAD seed. By processing the raw images for their fluorescent background and using machine learning to identify the T49-positive neuritic thread-like regions (Tau-positive zones), we quantified the number of T49-positive neuritic thread-like inclusions (Fig. 5). To quantify and separate our results between the two microfluidic compartments, (*i.e.* seeded and propagation compartments), we developed a Java computer program named the Cell Counter. Fig. 6 summarizes the main steps followed when using the Cell Counter. This computer program enabled us to automate and quantify Tau pathology in a neuronal microfluidic model.

### Optimization of experimental conditions and experimental analysis pipeline

**Figure 7.**
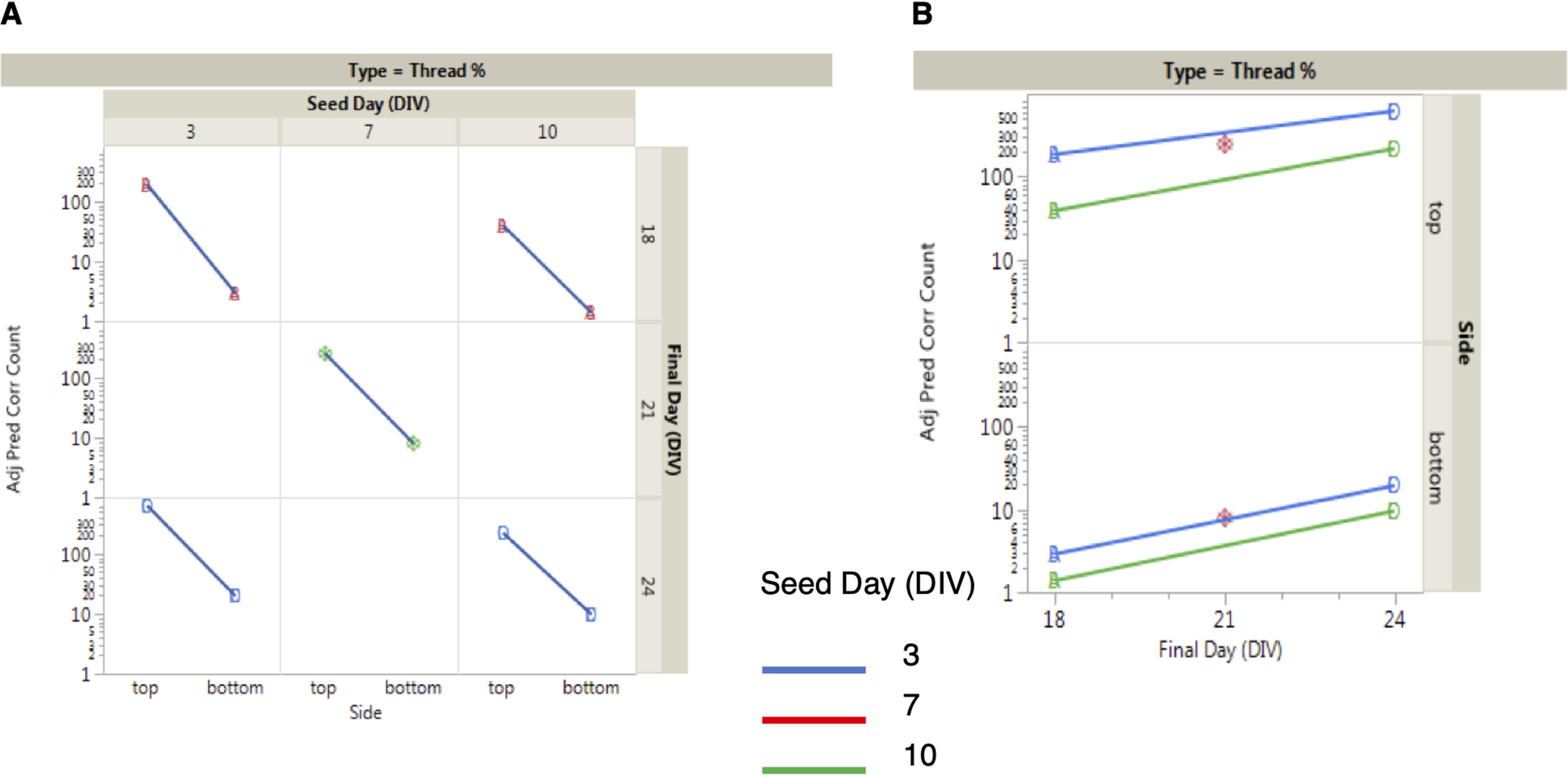
**Optimization of experimental conditions.** Seeding-side thread percentage (thread % = 100 × thread count/cell count) decreases as SD is postponed from DIVs 3 to 10 and increases as FD is postponed from DIVs 10 to 21 (*A*). Parallel lines between seeding and propagation side indicate that propagation side results are proportional to seeding side results across these assay conditions. Thread % is maximized with the SD/FD = 3/24 combination, although the risk of cell death is elevated with such a late FD, and thus SD/FD = 3/21 is operationally the optimal combination (*B*).

To optimize the experimental and analysis conditions, we ran a factorial experiment to assess the impact of seeding day, fixing day, and plate on the seeding and propagation side. RCNs plated in microfluidic devices in 6-well plates (one 2-compartment device per well) were seeded on DIV 3, 7, or 10 (seeding day, or SD) and fixed on DIV 18, 21, or 24 (final day, or FD). The standard conditions were SD = 7, FD = 21, and this along with SD/FD combinations 3/18, 3/24, 10/18, and 10/24 were tested on five 6-well plates containing microfluidic devices. Each plate had unseeded wells to assess the assay background conditions. The experiment was analyzed by mixed models analysis of variance. The results showed background levels did vary substantially across plates (>60% of total variation) and between seeding and propagation sides, indicating on-plate controls for background levels are required for this technology, and estimates of background levels should be subtracted from all seeded wells. The results showed that total cell counts decreased as SD is postponed from DIV 3 to DIV 10 and as FD is postponed from DIV 18 to DIV 24, especially on the propagation side, whereas thread-like inclusion counts increased. Thread % (thread-like inclusion count/total nuclei × 100) decreased as SD is postponed from DIV 3 to DIV 10 and increased as FD is postponed from DIV 18 to DIV 21, and strikingly the propagation side was a constant proportion of the seeded side (Fig. 7*A*). The results indicate that the length of time postseeding was the most important variable with SD = 3 and FD = 24 delivering the highest thread %; however, the risk of cell toxicity is elevated at FD = 24; therefore SD = 3 and FD = 21 was deemed to be operationally the best combination (Fig. 7*B*). However, because neurons in DIV 3 are not fully mature yet and the signal window calculated for SD = 7, FD = 21 is already sufficient to detect a high number of threads, we decided to use this condition for further experiments (33).

### Seeding and propagation of neuritic thread-like inclusions is concentration-dependent in the two-compartment microfluidic model

**Figure 8.**
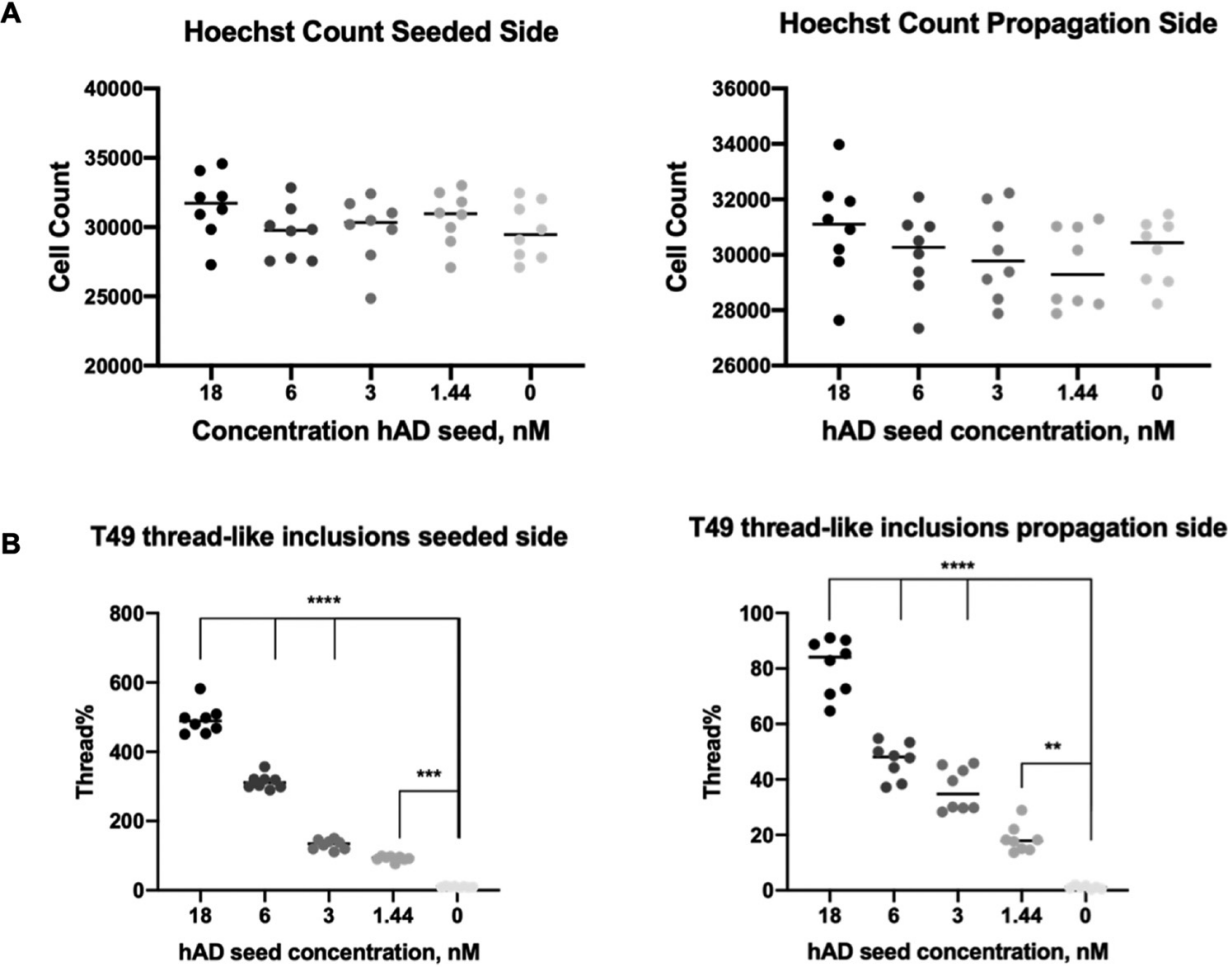
**Quantification of the number of neuritic thread-like aggregates demonstrates that propagation of the pathology in RCNs is concentration dependent.** Cell Counter quantification of the number of nuclei (Hoechst) and neuritic thread-like aggregates (T49) in both seeded (*A*) and propagation (*B*) compartments in a four-point CRC of the hAD seed in RCNs starting from 18 nm. Statistical evaluation was performed using one-way ANOVA followed by Dunnett's post test; *p* < 0.05, **, *** compared with the unseeded (0 nm) control. The results are represented as means ± S.E. of two independent experiments with eight replicates in total for each condition (thread % = 100 × thread count/cell count).

Having developed a tool to quantify the neuritic thread-like inclusions, we tested the robustness of the microfluidic assay under different cell culture conditions. For this purpose, we performed a dose-response treatment of the hAD seed on RCNs cultured in two-compartment microfluidic devices starting from 18 nm (Fig. 8). The treatment demonstrated a dose-dependent response in terms of the number of neuritic thread-like inclusions in both seeded and propagation compartments (Fig. 8, *A* and *B*). Noticeably, the amount of inclusions detected in the propagation compartment is proportional to the inclusions in the seeded compartment, as a direct effect of the seeding with hAD seed.

### The aggregation and propagation of Tau can be inhibited using a tool compound

**Figure 9.**
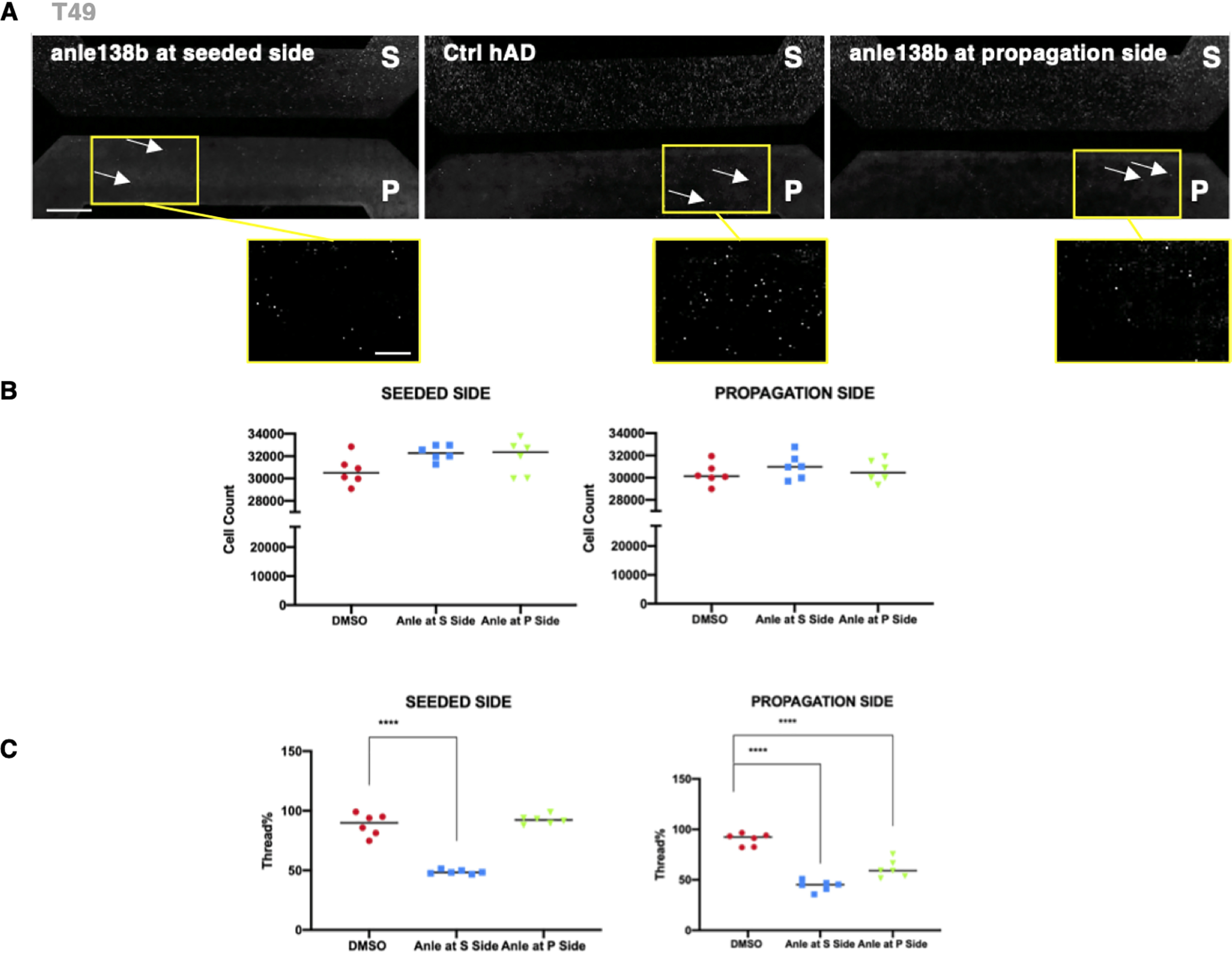
**Addition of a small molecule aggregation inhibitor to RCNs in the microfluidic model results in inhibition of both aggregation and propagation.** *A*, representative images of tiled montages of each two-chamber microfluidic device for each tested condition: anle138b added at the seeded compartment on the *left*, the DMSO hAD-seeded control (*Ctrl*) in the *middle*, and the anle138b added at the propagation compartment on the *right*, which demonstrates that the small molecule aggregation inhibitor anle138b had no effect on the number of Hoechst-positive cells and inhibited both the aggregation and propagation of neuritic thread-like T49 inclusions. *Figure bar*, 500 μm; *zoomed image bar*, 250 μm. *B*, there were no changes in the cell numbers between the DMSO control and the anle138b treatments when cells were counted for each microfluidic compartment. *C*, quantification of these results demonstrates a decrease of the number of T49-positive neuritic thread-like aggregates as a result of the anle138b treatment. Statistical evaluation was performed using one-way ANOVA followed by Dunnett's multiple comparison test (*p* < 0.05); the results are represented as means ± S.E. of two independent experiments with three replicates for each condition (thread % = 100 × Thread count/cell count). Zoomed images are indicated by *yellow squares*.

Having demonstrated that the count of neuritic thread-like inclusions can be modified by changing the cell culture environmental factors, such as the concentration of the seed, we tested whether we could pharmacologically reduce the number of inclusions in the propagation compartment. For this investigation we chose a compound, the di-phenyl-pyrazole anle138b that has been previously reported to inhibit protein aggregation in *in vitro* and *in vivo* systems (34). Using our 96-well RCNs cultures, we evaluated anle138b for a range of concentrations starting from 20 μm testing its ability to reduce the number of neuritic thread-like inclusions after hAD seeding (data not shown). The concentration that demonstrated the best inhibitory effect and no cytotoxicity was at 10 μm. We then tested anle138b under two different assay formats using our microfluidic culture model. First, we investigated whether the addition of both the compound and the hAD seed on DIV 7 in the seeded compartment would reduce the number of neuritic thread-like inclusions not only in this compartment, but also in the environmentally isolated propagation compartment (Fig. 9). Second, we investigated whether the addition of the compound on DIV 7 only to the propagation compartment would reduce the count of neuritic thread-like inclusions that propagate from the hAD-seeded culture (Fig. 9). The tiled images of the devices along with the count of Hoechst revealed that anle138b was not toxic to the cells over the course of the 2-week treatment period (Fig. 9, *A* and *B*). When anle138b was added to the seeded compartment it reduced the number of neuritic thread-like inclusions by ∼50% in both the seeded and propagation compartment, when compared with the DMSO-seeded controls (Fig. 9*C*). The effect of the treatment on the propagation side can be explained as the addition of anle138b at the seeded compartment reduced the number of neuritic thread-like inclusions formed and, consequently, the number of available inclusions that could propagate. Similarly, when anle138b was added to the propagation compartment, a ∼40% reduction in the count of neuritic thread-like inclusions was observed in that compartment when compared with the DMSO-seeded control (Fig. 9*C*) and no effect of the compound on the number of inclusions induced in the seeded compartment. This demonstrates that the compound did not passively diffuse to the seeded compartment. In summary, we have demonstrated that a tool compound inhibited aggregation and propagation of Tau in RCNs after seeding with purified seed extracted from human AD patients' brains.

### Enabling the use of a higher-throughput microfluidic platform to study the propagation of aggregated rodent Tau post hAD seeding

**Figure 10.**
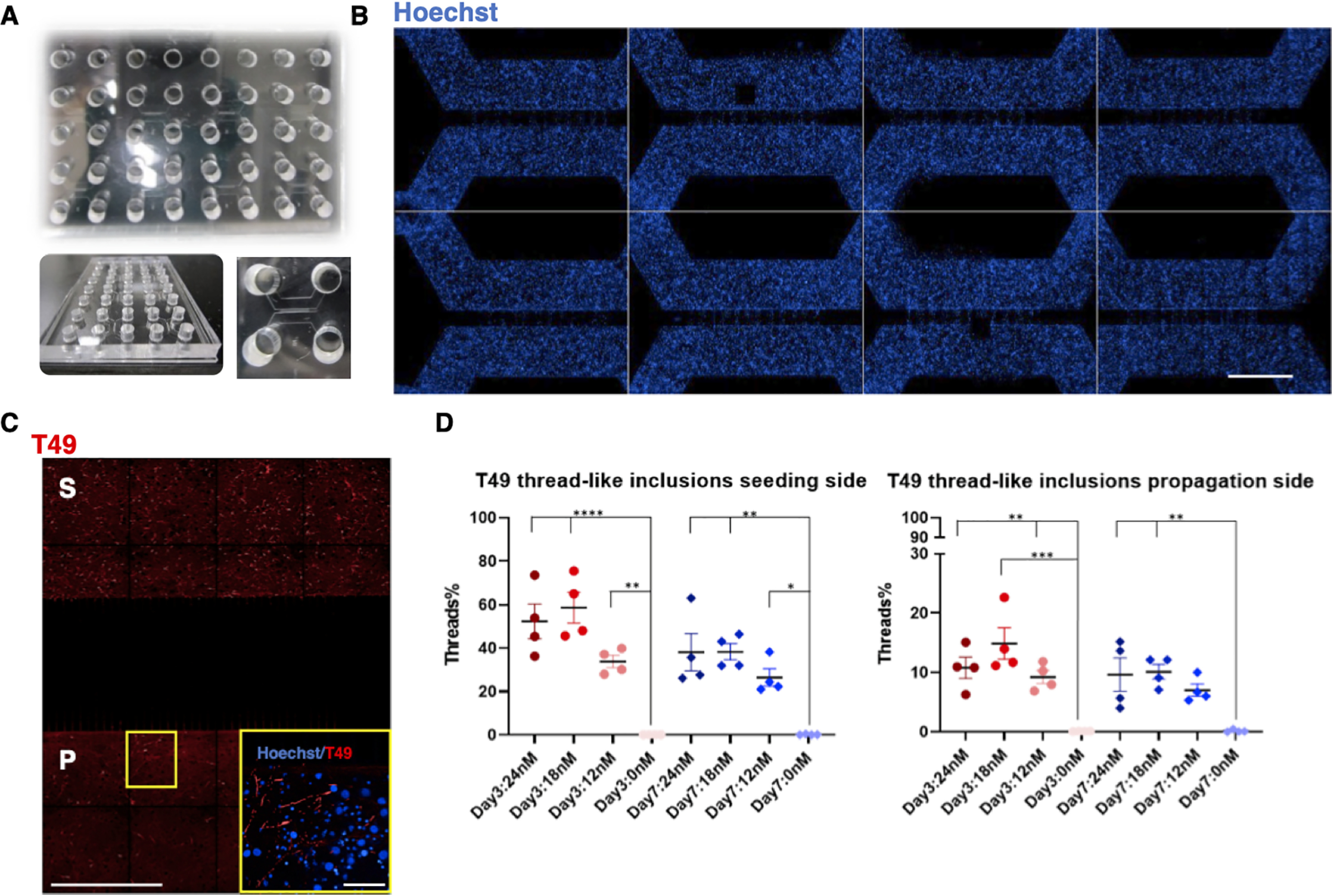
**Propagation of aggregated Tau in RCNs cultured in a novel higher-throughput microfluidic platform.** *A*, a typical unit of a higher-throughput microfluidic platform is PDMS-made and can support eight small two-compartment microfluidic devices in addition to eight isolated wells to enhance humidity when in culture. *B*, two units are used on a holder to obtain 16 two-compartment devices in the footprint of a standard 96-well plate, and RCNs were cultured in both compartments of this PDSM platform. *Bar*, 50 μm. *C*, when RCNs are seeded (*S*) with hAD seed, they demonstrate the neuritic thread-like inclusion morphology, and they propagate (*P*) the templated seed as revealed by the T49 staining (image tiled montage). *Figure bar*, 50 μm; *zoomed image bar*, 6.25 μm. *D*, quantification of the number of neuritic thread-like aggregates (T49) in both seeded and propagation compartments in a three-point CRC of the hAD seed in RCNs starting from 24 nm. Statistical evaluation was performed for each time point using one-way ANOVA followed by Dunnett's post test; *p* < 0.05, **, ***, **** compared with the unseeded (0 nm) control. There were no statistical differences between the single concentrations in DIV 3 and 7. The results are represented as means ± S.E. of two independent experiments with two replicates for each condition (thread % = 100 × thread count/cell count).

In our efforts to increase the number of replicates per experiment and to transform our microfluidic model to a higher-throughput assay format, microfluidic devices were designed so that the four wells of a two-chamber device matched the position of any 2 × 2 wells in a standard 96-well plate, thus increasing the number of experiments that could be performed simultaneously in the same footprint from 6 two-chamber devices to 16 two-chamber devices (Fig. 10*A* and Fig. S3*A*). This was done by creating a layout where each two-compartment microfluidic device had significantly smaller dimensions when compared with the commercially available ones (Fig. S3). Therefore, cell densities and volumes of the culture medium were optimized, leading to the successful culture of RCNs, following similar protocols as described above. The cells were seeded on two different days, DIV 3 and DIV 7, and fixed on DIV 21. The count of neuritic thread-like inclusions revealed that all concentrations of hAD seed tested both on DIV 3 and DIV 7 were able to induce Tau aggregation and propagation in this model with a peak at 18 nm in both cases, consistent with the results obtained with the commercial two-chamber devices (Fig. 10*D*).

The counts of neuritic thread-like inclusions in these higher-throughput microfluidic platforms were similar to those obtained in the commercial standard two-compartment microfluidic devices. This observation, combined with the increased number of replicates and reduced quantity of seeding material required, supports the use of this microfluidic plate platform as a higher-throughput tool to study the propagation of Tau and develop therapeutic strategies.

## Discussion

In the present study, we observed that human AD seed extracted from AD patients' brains can act as a template that induces aggregation of endogenous Tau in RCNs and that leads to further propagation between neuronal populations in an *in vitro* microfluidic system. Using Sarkosyl extraction and Western blotting, we demonstrated that hAD seed induces aggregation of endogenous Tau when applied to RCNs. Microfluidic devices have been previously used in the literature as an *in vitro* model system to study propagation of insoluble protein aggregates (27, 32). Their design renders them an ideal method to study effects among isolated cellular populations because cells are physically separated by microgroove barriers. Moreover, fluidic isolation between the compartments ensures that the transmission of the aggregates happens because of cellular mechanisms and not by passive diffusion. By using a unique plate assay format with two-compartment microfluidic devices, we demonstrated that hAD seed not only causes aggregation of endogenous Tau in RCNs, but it also results in the propagation of this endogenously generated pathological Tau. Although our system supports synaptically connected neurons between the two compartments, we cannot exclude that propagation could occur via retrograde transport of aggregates rather than synaptic transmission, consistent with that observed by Wu *et al*. (23); directionality of spread could be confirmed in future using unidirectional microfluidic devices that allow axons to grow just in one direction. Our study shows the propagation of aggregated Tau in *in vitro* microfluidic devices in a quantitative and highly reproducible assay format after seeding with hAD seed. Furthermore, we observed inhibition of propagation when we intervened pharmacologically by using anle138b, a compound that has been previously reported to inhibit oligomer formation and disease progression in *in vitro* and *in vivo* models of synucleinopathies and prion disease (35).

In our *in vitro* propagation system, a nonrecombinant form of seed was used to induce aggregation in RCNs. This enhances the argument for more native and disease relevant models because neither overexpression of Tau nor mutations on the Tau protein are a cause of AD (36). Another interesting finding is that the seeding on DIV 3 resulted in higher numbers of neuritic thread-like inclusions in both seeded and propagation compartments compared with seeding on DIV 7. It is possible that neurons at DIV 3 exhibit enhanced uptake or reduced clearance of hAD seed compared with DIV 7, which could account for their enhanced response to seeding. Additionally, Wu *et al*. (27) observed that neuronal activity enhances Tau propagation and Tau pathology *in vitro* and *in vivo*; therefore differences in neuronal activity between DIV 3 and DIV 7 could account for the different responses to seeding observed at these time points. Importantly, the concentration of the hAD seed used in our experiments did not result in acute cellular toxicity as revealed by nuclei count. Seeded presynaptic neurons were viable when transmitting the pathological forms of Tau to postsynaptic neurons, suggesting that transmissibility of the inclusions is an event occurring prior to neuronal death. This finding is consistent with recent research suggesting that misfolded Tau does not directly cause neuronal death but targets and impairs axonal transport and synaptic loss first (37). Furthermore, at a more clinically relevant level, it has been shown that the spreading of Tau preceded tangle formation in the brain (38). While using the commercially available devices, we occasionally observed Hoechst-positive cells within the microgrooves. Although we control for purity of the neuronal cultures, we expect to find some glia cells. Microglia cells being smaller than neurons could potentially travel through the microgrooves and therefore could facilitate the spread of aggregated Tau and is something to be considered in future experiments (39).

anle138b has previously been described *in vitro* and *in vivo* for its effects against oligomer formation; in mouse models of prion disease and PD, anle138b inhibited oligomer accumulation, neuronal degeneration, and disease progression (35). Recently, early treatment with anle138b (before significant neuronal loss) reversed the motor impairments in a mouse model of PD demonstrating, that early inhibition of α-synuclein aggregation can rescue the dopaminergic dysfunction and motor features in a mouse *in vivo* model (40). Our data show an inhibitory effect of anle138b on both aggregation and propagation in an *in vitro* model of Tau aggregation and propagation in microfluidic devices. It is possible that some of the aggregates that we detect in the propagation side of all our devices are still inside the axons of neurons plated in the seeding side. However, when we used anle138b at the propagation side of the commercial devices, we detected a decrease in newly formed T49 inclusions. Because anle138b is inhibiting the formation of new inclusions but not the disaggregation of the already formed inclusions, we can infer that the majority of inclusions we count at the propagation side belong to the neurons plated in that compartment. anle138b has been shown not to affect Tau expression, Tau ubiquitination, or Tau autophagy, and its primary mechanism of action seems to be inhibition of oligomers formation in a wide range of amyloidogenic peptides and proteins, by interfering with the formation of ordered β-sheet structures (34). The broad spectrum of activity of anle138b in models of different neurodegenerative diseases suggests that pathological oligomers in such diseases may have common structural features, and compounds that target structure-dependent (and not protein-dependent) epitopes on such oligomers may have the potential to treat multiple protein aggregation diseases.

Microfluidic devices offer an ideal platform to study neuronal connectivity and spread of Tau pathology, and the number of publications appeared in recent years (7, 23–28, 37) supports their utility as an *in vitro* model of AD to test intervention against neuronal cell death with loss of synapses and neuronal network, which is one of the main pathophysiological characteristics of AD. Moreover, the architecture of microfluidic devices offers the potential to create even more complex *in vitro* systems, which may better recapitulate the multifactorial nature of neurodegenerative diseases. The interplay between neuroinflammation and neurodegeneration has recently become clearer because it has now become established that the pathological effects in AD are cell- and non–cell-autonomous and occur in the context of neurovascular or glioneuronal units (41). Microfluidic systems and 3D cultures now offer the opportunity to recapitulate these effects in experimental models and present themselves as ideal systems to study therapeutic interventions. Toward the efforts of producing more disease relevant experimental models, microfluidic devices can also support the growth of human induced pluripotent stem cell-derived neuronal cultures that can be seeded and transmit seed induced pathology (42). In an effort to transform our system to a higher-throughput assay format, we used microfluidic plates allowing the user to handle 16 two-compartment devices simultaneously, thus increasing the throughput compared with commercially available microfluidic devices. We are now testing microfluidic plates that can host 80 two-compartment devices in the same footprint of a standard well plate (where open wells match the format of a 384-well plate), providing further increase in throughput and assay miniaturization.

In conclusion, we have developed and validated a quantitative method and a robust cellular model of human sporadic tauopathies using RCNs seeded with hAD Tau to quantify formation and propagation of Tau inclusions and test the effect of inhibitor molecules, which can be further miniaturized for high-throughput screening and could be adapted to monitor propagation of any prion-like disease.

## Experimental procedures

### Primary cultures in microfluidic devices

Two-compartment 6-well plate format microfluidic devices with 450-μm-long microgroove barriers (Xona Microfluidics, SND450) were plasma-treated (Henniker Plasma Systems) for 45 s at 50% power and attached to plasma-treated 6-well glass-bottomed plates (Cellvis, PO6-1.5HN); 96-well format microfluidic devices were plasma-treated and attached to plasma-treated coverslips (50 × 75 mm (1.96 × 2.95 inches), thickness no. 1, Tedpella, catalog no. 260462). All microfluidic compartments and glass surfaces were coated with poly-d-lysine (Sigma–Aldrich) at 0.1 mg/ml. RCNs were cultured from E18 Sprague–Dawley rat embryos (Charles River Laboratories) in the 6-well plate microfluidic devices at a density of 4 × 10^4^ cells/μl/compartment and in the 96-well plate microfluidic devices at a density of 2.5 × 10^4^ cells/μl/compartment. The cultures were maintained at 37 °C and 5% CO_2_ up to 21 days (unless stated otherwise), and the culture medium was changed every 4 days.

### Human AD seed preparation

The purification of Tau from hAD brains was adapted from Greenberg and Davies (29) with modifications. Human brain tissues from AD patients were obtained from Manchester Brain Bank and King's College London Brain Bank and characterized by AlphaScreen, as previously described (43) before purification. The pool of cortical tissues used were obtained from ∼20 patients with Alzheimer's disease, modified Braak (Brain Net Europe) stage 6 with moderate amyloid angiography. The average age was 71 years, and the gender was mixed male and female. The samples chosen for the preparation were those with the highest levels of AT8-positive Tau (0.5 μg/ml) as determined by AlphaScreen. For each purification, the tissue was thawed, the white matter was dissected out, and 100 g of cortical gray matter was homogenized using an Ultra Thurrax (IKA T25, 25,000 rpm, 10 min) in 400 ml of Dulbecco's Phosphate Buffered Saline (DPBS) supplemented with Complete protease inhibitor tablet (Roche) and centrifuged at 10,000 g for 10 min at 4 °C. The pellets were re-extracted twice using the same buffer conditions as the starting materials, and the supernatants from the three extractions were filtered through a Kim wipe and pooled. 30% Sarkosyl was added to the pooled supernatant for a final 1% concentration. The sample was incubated in a glass bottle, shaking at room temperature for 1 h on a flat rotating shaker at a medium speed. After Sarkosyl extraction, the sample was centrifuged at maximum speed (45,000 rpm, 158,000 g) for 60 min at 4 °C in an Optima XPN-80 ultracentrifuge. The resulting 1% Sarkosyl-insoluble pellet was washed once in PBS/Complete, then resuspended in Tris 50 mm, pH 7.4, containing Complete (50 μl Tris/g gray matter) and sonicated with ∼20 1-s pulses (40% amplitude, Soniqa Q125 sonication probe) and named “hAD seed.” Subsequently, Tau concentration of the hAD seed was determined using AlphaScreen as described previously (43). When used to treat neurons, the hAD seed stock was diluted in media to the appropriate concentration.

### Seeding of primary RCNs with hAD seed in microfluidic devices

All animal procedures were performed in accordance with the Animals (Scientific Procedures) Act 1986 and were approved by the Eli Lilly Animal Welfare Board. RCNs were plated at a density of 40,000 cells/μl in each compartment of two-compartment microfluidic devices plasma-bonded to glass-bottomed 6-well plates and were seeded with hAD seed on DIV 7; the hAD seed was prepared at 2× in culture medium, sonicated for 60 1-s pulses on ice at 20% amplitude on a Soniqa Q125 sonication probe, and filter-sterilized using a 0.2-μm syringe filter. The seed was added at the seeded compartment of the devices. Culture medium was aspirated and replaced with the hAD seed. Passive diffusion of the seed was prevented by a hydrostatic pressure barrier created by the volume medium gradient between the seeded and propagation compartment (respectively, 80 μl *versus* 200 μl). The devices were kept attached on the glass-bottomed 6-well plates even after fixation of the cells, and this plate format was maintained for high-content imaging to detect aggregated pathological Tau at the propagation side. The protocol followed for microfluidic devices the 96-well plate format was the same as above, with the following modifications in the cell plating density and in the volumes used: RCNs were plated in each compartment at a (reduced) density of 25,000 cells/μl, and the volume medium gradient was maintained with (reduced) volumes of 30 μl *versus* 60 μl.

### Sarkosyl fractionation and Western blotting of RCNs following seeding

RCNs were plated at 1.7 × 10^6^ cells/well, 2 ml/well, in 6-well plates (Corning^®^ BioCoat™ poly-d-lysine 6-well clear flat-bottomed tissue culture-treated multiwell plate, catalog no. 354413). Neurons were seeded with hAD seed on DIV 7; seed was not removed, and only half-media changes were done biweekly until harvesting. On DIV 21, neurons were harvested and lysed in radioimmune precipitation assay buffer (Sigma, catalog no. R0278) supplemented with 0.5 mm phenylmethylsulfonyl fluoride, 2 mm sodium orthovanadate, phosphatase inhibitor mixture 3 (Sigma, catalog no. P0044), phosphatase inhibitor mixture 2 (Sigma, catalog no. P5726), and Complete mini protease inhibitor mixture Tablet (Roche, catalog no. 11836153001). The lysates were kept frozen at −80 °C.

For the Sarkosyl fractionation, the lysates were thawed and centrifuged at 13,000 × *g* for 10 min at 4 °C to clear cell debris, and the supernatant protein content was determined by BCA assay. After normalizing to the lowest protein concentration, the samples were named total lysates (TLs). An aliquot of TL was retained for further analysis. 30% Sarkosyl aqueous solution (Sigma, catalog no. 61747) was added to the TLs for final 1% and incubated with shaking for 1 h at room temperature at medium speed. TLs were centrifuged for 60 min at 100,000 × *g* (48,000 rpm) at 4 °C in an Optima TLX 55 ultracentrifuge. The pellets were washed twice with 0.5 ml of radioimmune precipitation assay buffer, centrifuged 5 min at 13,000 × *g*, and resuspended in 1/10th of the original volume spun of 50 mm Tris, pH 7.4, supplemented with Complete tablet (Roche) and 1 mm phenylmethylsulfonyl fluoride. The pellets were sonicated ∼20 1-s pulses, 40% amplitude until completely dispersed, and frozen at −80 °C.

For the SDS-PAGE–Western blotting, Sarkosyl-insoluble pellet samples were prepared with 4× lithium dodecyl sulfate (LDS) NuPAGE sample buffer containing 5% β-mercaptoethanol, heated to 95 °C for 10 min, and loaded onto 8% Bis-Tris NuPAGE midi gels. The gels were run in the Invitrogen Xcell SureLock midi gel apparatus using 1× NuPAGE MOPS SDS running buffer. The gels were run at 200 V until the dye front reached the bottom of the gel. Proteins were transferred to Amersham Biosciences Hybond nitrocellulose membranes, which were blocked and incubated with primary and secondary antibodies. Primary antibodies used were T49 (1:1000) (Millipore, catalog no. MABN827), AT8 (Ser(P)^202^/Thr(P)^205^) (1:1000) (Thermo Fisher, catalog no. MN1020), mouse anti-3R Tau (1:1000) (Millipore, 05-803), rabbit anti-4R Tau (1:1000) (Cosmobio, catalog no. CAC-TIP-4RT-P01), and glyceraldehyde-3-phosphate dehydrogenase (Amersham Biosciences, catalog no. AM4300). Secondary antibodies used were anti-mouse IgG HRP (1:10,000) (Cell Signalling, catalog no. 7076), anti-rabbit IgG HRP (1:10,000) (GE Healthcare, catalog no. NA934), and sheep anti-mouse IgG HRP (1:10,000) (GE Healthcare, catalog no. NXA931). All membranes were incubated in West-Femto detection reagent, apart from glyceraldehyde-3-phosphate dehydrogenase detection, where membranes were incubated in West-Dura detection. Images were acquired using ImageQuant LAS 4000 Mini.

### Immunocytochemistry

RCNs were washed twice with DPBS and then fixed with prechilled (−20 °C) methanol to extract soluble proteins for 15 min (30). Afterward the cells were washed three times with DPBS and were kept at −4 °C until use. The cells were blocked and permeabilized for 1 h at room temperature with 0.1% Triton Odyssey blocking buffer (LI-COR). After blocking, the cells were incubated with primary antibodies diluted in 0.1% Triton Odyssey blocking buffer overnight at −4 °C while resting on a shaker with slow speed. The following day the cells were washed three times with DPBS (100 μl/compartment) and then were incubated with the secondary antibodies and Hoechst diluted in 0.1% Triton Odyssey blocking buffer for 1 h in the dark at room temperature. HCI was performed using an Opera Phenix system (PerkinElmer) with a 20× 0.8 N/A air objective, water-immersed 40× objective, and Operetta with a 20× 0.4 N/A air objective.

The primary antibodies used are rabbit anti-MAP2 (1:1000) (Millipore, catalog no. AB5622); rabbit anti-MAP1B (1:1000) (Thermo Fisher, catalog no. PA5-78052), mouse anti-MAPT (DA9) (1:1000) kindly donated by Peter Davies (44), and mouse anti-T49 (1:2000) (Millipore, catalog no. MABN827). The secondary antibodies used are Alexa Fluor 594 anti-rabbit IgG (1:1000) (Invitrogen, catalog no. A110112), Alexa Fluor 488 anti-mouse (1:1000) (Invitrogen, catalog no. A11001), and Alexa Fluor 647 anti-mouse IgG1 (1:000) (Invitrogen, catalog no. A21240).

### HCI analysis with Harmony software

For each microfluidic device a total of 153 image fields were captured using a 3% field overlap. For each image field a total of 10 *z*-stack planes was used, from −8 to 2.8 μm. To acquire a tiled image for each microfluidic device, the image fields were stitched together and maximum projected with an advanced flat-field correction. Hoechst staining was used to label all nuclei. The raw T49 images were filtered using the sliding parabola method (threshold at 200) to smooth the fluorescent background. For each image field, all the stack planes were maximum projected. Machine learning was then used to identify the T49-positive staining zones from the unspecific diffused background that is observed in the absence of the hAD seed. In particular, the Harmony software building block used for the machine learning is called “find texture region,” which allows a supervised texture segmentation. In the training step the user selects some examples of texture classes (*e.g.* Tau-positive region or background), and the software divides each image into two regions accordingly with the training, each being similar to a particular example class and different from the other example classes. Within these T49 Tau-positive defined areas, the number of the Tau inclusions was counted using the building block “find image region” and plotted as the main assay parameter. The data are extracted per well and object. The data were plotted using GraphPad Prism 8.3.0 (San Diego, CA, USA) or JMP^®^ 12.1.0 (SAS Institute Inc., Cary, NC, USA).

### HCI analysis with in-house developed Cell Counter program

For the analysis of cell counts under different conditions, an interactive computer program was created. The program, written in Java, was designed to display the *x*-*y* locations of cells counted by the microscope in a simple *x*-*y* plot. It allowed the “top” and “bottom” regions of the microfluidic device to be defined, either by an automated algorithm detecting density or interactively by the user. To implement automatic detection of regions, a procedural algorithm was used. The flow was as follows: Step 1: Determine the average density of points across the image. Step 2: Starting from the bottom of the image, move up the image and determine where the moving average of density in a strip 10% of the width of the image goes from below average to above average. This marks the start of the bottom cell. Step 3: Continue upwards until a drop in average density is observed. This marks the end of the cell. Step 4: Repeat steps 1 and 2 to find the top cell limits. Step 5: For each cell, move left and right until the density drops below the average threshold to find the left and right sides of the cells. Some manual tuning of the exact threshold relative to the average density was done to find values that worked best across a range of images. Once the region was defined on a control sample (the unseeded devices), the program counted points within the same region for other conditions and produced aggregated statistics (total count and point density within each area) stratified by well position and treatment condition, for further analysis. The program also annotated all of the points in the original input file with a simple “top,” “bottom,” or “no” label indicating in which region every point lay. This information was also used for further validation. The combined codes can be found in the supplementary information, and the codes can be run using Java.

### Treatment of RCNs with anle138b

The anle138b compound (35) was synthesized in-house, a stock concentration of 10 mm in DMSO was prepared, and aliquots stored at −20 °C. The compound was diluted to 10 μm in culture media and added to the cells on DIV 7.

## Data availability

Most of the data described in the article are contained within the article or in the supporting information. Any data not shown can be requested from the corresponding author: bose_suchira@lilly.com.

## Supplementary Material

Supporting Information
