# Supplementary material for: Quantitative propagation of assembled human Tau from Alzheimer's disease brain in microfluidic neuronal cultures
Source: J Biol Chem. 2020 Jul 22;295(37):13079–93. doi: 10.1074/jbc.RA120.013325 (PMC7489902; doi:10.1074/jbc.RA120.013325)
Supplement: Supporting Information [file supp_295_37_13079__index.html]

Quantitative propagation of assembled human Tau from Alzheimer's disease brain in microfluidic neuronal cultures — Quantitative propagation of assembled human Tau — Quantitative propagation of assembled human Tau from Alzheimer's disease brain in microfluidic neuronal cultures — Quantitative propagation of assembled human Tau — Supporting Information 

# Quantitative propagation of assembled human Tau from Alzheimer's disease brain in microfluidic neuronal cultures

## Supporting Information

- Supplementary Figures and Legends - Supplementary Figures and Legends
- Supporting Information (to be published online) - Combined codes for running cell counter program using java
