# Supplementary material for: Quantitative propagation of assembled human Tau from Alzheimer's disease brain in microfluidic neuronal cultures
Source: J Biol Chem. 2020 Jul 22;295(37):13079–93. doi: 10.1074/jbc.RA120.013325 (PMC7489902; doi:10.1074/jbc.RA120.013325)

SUPPLEMENTARY FIGURES

Figure S1. hAD seed concentration response curve in RCNs in a 96-well assay format. High content imaging reveals a concentration-dependent increase in the number of T49-positive neuritic thread-like inclusions resulting from addition of increasing concentrations of hAD seed. The parameters considered were the count of T49 neuritic-like threads and the hAD seed dose response. (A). AD seed concentrations above 0.75 µg/ml (18 nM) induced occasional cellular toxicity as demonstrated by the count of Hoechst nuclei. Bar, 100 µm (B). The number of T49-positive neuritic thread-like inclusions increased proportionally to the concentration of hAD seed (C; graph plotted in log10 scale). Statistical evaluation deemed that 0.75 µg/ml (18 nM) of AD seed produced the best signal window of seeding. Image acquisition with Operetta, 8 replicates for each condition. (Thread % = 100 * Thread count/cell count).

Figure S2. Time course of endogenous tau expression in RCN. The expression of total Tau (DA-9), 3R and 4R Tau isoform was followed over time in RCN. Western blot experiments show that total tau expression, as well as 3R tau expression, increases after a week and stays stable until it decreases at DIV21. 4R tau expression increases over time and peaks at DIV15 before it starts decreasing. These results show that at the time of seeding (DIV7) both 3 and 4R tau isoforms are expressed by RCN.

Figure S3. Microfluidic platform devices for enabling transformation of microfluidic assay model to a higher throughput assay format by increasing the number of experimental replicates. The higher throughput platform microfluidic devices (A) have significantly smaller dimensions (B) when compared to the commercially available devices from Xona (C). Two 8 two-chamber devices can be slotted into a holder that has the same footprint of a 96 well plate (i.e. ~128 mm x ~86 mm), thus obtaining 16 two-chamber devices in comparison when using commercially available ones where only 6 two-chamber devices can be slotted in the same footprint, thus increasing the throughput.

SUPPLEMENTARY INFORMATION

See supplementary file for the combined codes. To run the combined codes program requires a working java runtime (version 8 or higher) to be installed.

SUPPLEMENTARY FIGURES

Supplementary Figure 1

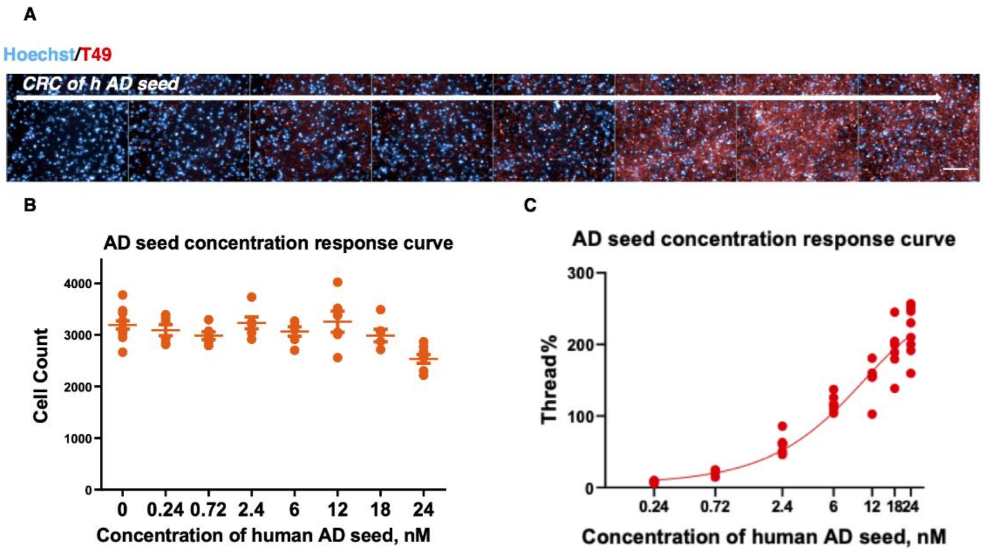

Supplementary Figure 2

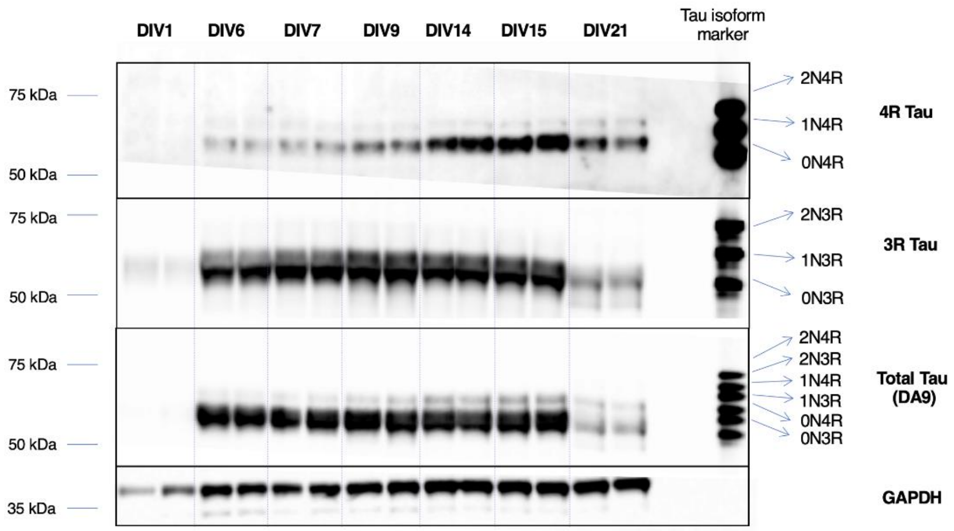

Supplementary Figure 3

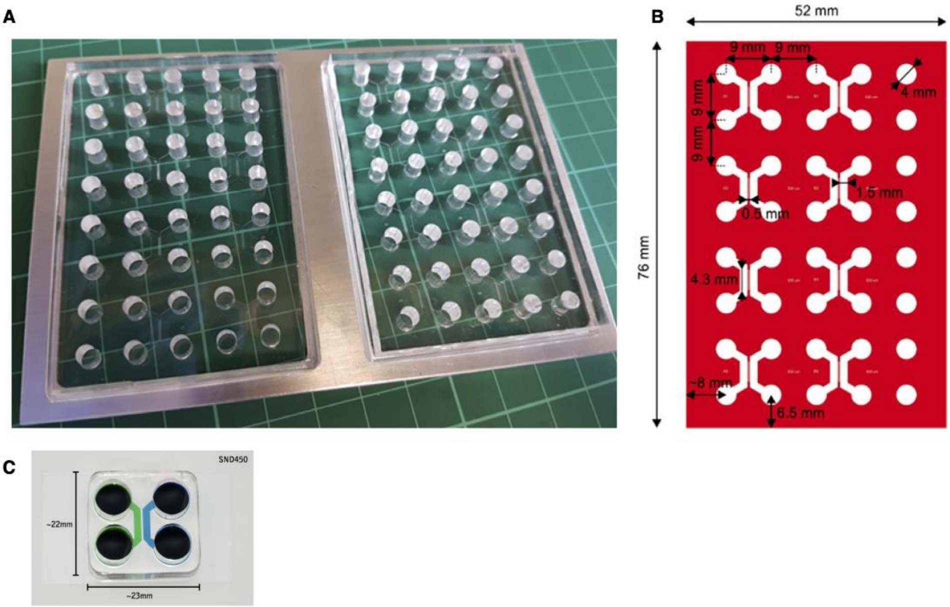

Supplement: Supporting Information [file supp_RA120.013325_159277_3_supp_data_566788_qdrnrn.pdf]
